# Rejecting Chaotic Disturbances Using a Super-Exponential-Zeroing Neurodynamic Approach for Synchronization of Chaotic Sensor Systems

**DOI:** 10.3390/s19010074

**Published:** 2018-12-25

**Authors:** Dechao Chen, Shuai Li, Qing Wu

**Affiliations:** 1 School of Computer Science and Technology, Hangzhou Dianzi University, Hangzhou 310018, China; chdchao@hdu.edu.cn; 2 Department of Computing, The Hong Kong Polytechnic University, Hung Hom, Kowloon, Hong Kong, China

**Keywords:** zeroing neurodynamic, recurrent neural networks, chaos, sensors, chaotic disturbance rejection, fast synchronization, 62G35, 92B20, 93B51

## Abstract

Due to the existence of time-varying chaotic disturbances in complex applications, the chaotic synchronization of sensor systems becomes a tough issue in industry electronics fields. To accelerate the synchronization process of chaotic sensor systems, this paper proposes a super-exponential-zeroing neurodynamic (SEZN) approach and its associated controller. Unlike the conventional zeroing neurodynamic (CZN) approach with exponential convergence property, the controller designed by the proposed SEZN approach inherently possesses the advantage of super-exponential convergence property, which makes the synchronization process faster and more accurate. Theoretical analyses on the stability and convergence advantages in terms of both faster convergence speed and lower error bound within the task duration are rigorously presented. Moreover, three synchronization examples substantiate the validity of the SEZN approach and the related controller for synchronization of chaotic sensor systems. Comparisons with other approaches such as the CZN approach, show the convergence superiority of the proposed SEZN approach. Finally, extensive tests further investigate the impact on convergence performance by choosing different values of design parameter and initial state.

## 1. Introduction

In 1963, Edward Lorenz [1] started to introduce and report the research on chaotic attractor. Since this inspiring work, extensive research on the chaos handle and synchronization of sensors has been investigated and developed for industrial electronics [2,3,4,5,6]. The technology of chaotic sensor includes two significant parts, i.e., information acquiring as well as information processing. Due to the complexity and nonlinearity, the investigation of chaos synchronization of advanced sensor systems for rejecting chaotic disturbances is a tough issue [7,8,9,10]. Specifically, as one of the attractive phenomena in the sensor processing, the chaotic synchronization of sensor systems has been a heated issue that researchers have focused on recently [11,12,13]. For the real-time chaotic synchronization of sensor systems, the procedure is that two chaotic systems adapt the provided properties of the motions to usual behavior by converging in real-time *t* [14]. In other words, starting from random or different initial states, all the real-time states of the response, i.e., slave chaotic sensor systems are forced to track all the real-time states of the drive, i.e., master chaotic sensor systems. The synchronization of chaotic sensor systems has numerous practical applications, such as secure communications [15,16], sensor systems [17], finance systems [18] and electronic systems [19], ecological systems [20], and many other engineering systems [21]. Moreover, various feasible real sensing applications in the fields of sensing and chaotic sensor systems have been reported [22,23,24,25,26,27,28,29]. For example, a novel circuit implementation of the chaotic Lorenz system was introduced in [22]. Note that the corresponding chaotic behavior of the circuit systems closely conforms to those results predicted by the numerical experiments. The synchronized chaotic systems were finally utilized in the communications. Teodorescu [23] detailedly investigated a mechanism that describes the high sensitivity and selectivity in a sensor neural network system. In [23], several new sensor concepts were proposed and introduced by the measurement on the basis of chaos. By utilizing the nonlinear dynamic chaotic sensor system, the high sensitivity measurement was effectively obtained. In [24], a novel detection system of a metal detector was developed by utilizing a chaotic system-duffing oscillator. Karimov et al. [25] presented the analysis of two kinds of signals, which illustrated that chaotic signals show high resistance to crosstalk, and were less influenced by the transmission loss compared to chirp signals. Tlelo-Cuautle et al. [26] novelly proposed an elegant system with the open curve of equilibrium points, of which the chaotic oscillator was implemented via the field-programmable-gate-array (FPGA). In [27], the authors developed a new multilayer perceptron (MLP), which was implemented by effectively utilizing an FPGA to forecast experimental-chaotic time-series. In [15], Yang and Zhang novelly introduced a global chaotic synchronization approach for identical systems, and successfully applied it to secure communication. In addition, Naderi and Kheiri [16] investigated the exponential-chaotic synchronization of the system together with the successful application in secure communication. A novel synaptic weight update learning-rule of Hermite-neural-network was introduced in [21]. Chen et al. [30] novelly investigated the hybrid synchronization feature in the array of coupled-chaotic systems. Moreover, numerical, analog and digital circuit models were detailedly investigated and presented in [19] with the 3-dimensional, continuous and autonomous new chaotic system.

Extensive research has been done for chaotic synchronization of sensor systems by employing and utilizing the effective approaches up to now, such as the neurodynamic approach [31,32], the active control approach [14,33], the sliding mode control approach [2], the model predictive control approach [34], and the adaptive backstepping control approach [35]. For instance, Li et al. [31] presented a simple controller designed by the conventional zeroing neurodynamic (CZN) approach for synchronization of chaotic systems. Moreover, Ahmad et al. [14] studied and investigated the global-chaotic synchronization issue for two identical as well as nonidentical chaotic systems via novelly utilizing a linear-active-control (LAC) approach. In addition, Zhang and Liu [34] novelly proposed a robust model predictive control approach to address synchronization of the discrete-time-chaotic systems with polytopic-model-uncertainties. Lin et al. [35] proposed a systematic approach for developing, as well as neural-adaptive backstepping-control of the chaotic system with uncertainties. Li et al. [36] novelly developed a method for the impulsive lag synchronization of chaotic systems.

Due to the advantages in terms of distributed storage, parallelism and easy implementation by the hardware, the neurodynamic approach has been served as a prior alternative for chaotic synchronization of sensor systems by researchers as well as engineers [37,38,39,40,41,42,43,44,45]. As the new branch of recurrent-neural-networks (RNNs) [46,47,48,49], various neural network models designed by the CZN approach have been developed as effective tools for the online engineering issues handling, as well as real-time synchronization of chaotic systems [50,51,52,53,54,55,56]. For instance, Zhang et al. [53] detailedly investigated the control issues of three kinds of chaotic systems by combining the CZN approach as well as the gradient neurodynamic approach to design an effective controller. Moreover, an effective stabilization control method of the hyper-chaotic system with only-one control-input was investigated in [55] by using a neurodynamic approach. Jin et al. [56] novelly proposed a controller-design approach, for tracking-control of a modified Lorenz chaotic system with the division by zero problem conquered.

By utilizing the information of the time derivative, extensive works for different real-time engineering problems as well as control and synchronization of chaotic sensor systems were developed by leveraging the CZN approach. It was theoretically proved that the associated models or controllers designed by the CZN approach can find the theoretical online solutions with global-and-exponential convergence property. However, such a convergence property may still not be sufficient for a strict real-time requirement in a complex environment, such as real-time secure communication systems and radar communication systems [57,58]. In addition, as the complexity of systems increases, the computational scale would become intensively large, and the synchronization accuracy would become particularly low while the real-time capability of chaotic systems needs to be considered in some practical applications. For instance, in [31], the authors developed a simple controller on the basis of the CZN approach. It was proven in [31] that resultant synchronization-error exponentially converges towards zero without considering disturbances by using such a controller. However, the low synchronization accuracy may occur if such a controller is applied in the real-time synchronization of chaotic systems within a short synchronization duration, which finally leads to the failure of synchronization process and chaos behavior.

**Figure 1:**
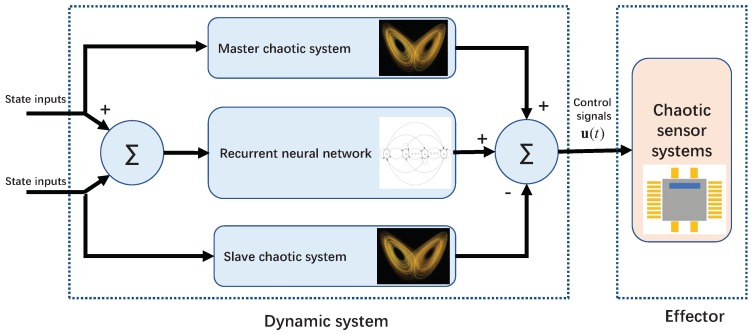
Zeroing neurodynamic architecture for designing the controller of chaotic sensor systems. Note that the control signals u(t) are transmitted to the system effectors (i.e., the slave chaotic sensor systems).

Being superior to the above-mentioned works on the basis of the CZN approach, this paper introduces and develops a novel super-exponential-zeroing neurodynamic (SEZN) approach and its associated controller. Quite different from the CZN approach with exponential convergence property, the controller designed by the proposed SEZN approach inherently possesses the advantage of super-exponential convergence, which makes the synchronization process faster and more accurate. Theoretical analyses rigorously prove the stability and convergence superiorities in both faster convergence rate as well as lower error bound within the synchronization duration. To the best of the authors’ knowledge, the SEZN approach as well as its associated controller with the outstanding super-exponential convergence property for the chaotic synchronization of sensor systems have not been investigated in the existing research. This work considers the chaotic issue related to sensor systems. It is a critical issue and some sensors fail exactly due to the chaotic disturbances. This is the motivation for us to develop a theoretical model to harness this issue. The zeroing neurodynamic architecture for designing the controller of chaotic sensor systems is presented in Figure 1 for better understanding the main principle.

The remaining part is structured as follows. The formulation of synchronization between two chaotic systems is presented as preliminaries, and the corresponding controllers designed by the SEZN approach as well as the CZN approach are presented in Section 2. In Section 3, rigorous theoretical analyses on the controller designed by the proposed SEZN approach are presented. Section 4 shows simulation studies including three synchronization examples, comprehensive comparisons and extensive tests. Section 5 concludes this paper with future directions. Before ending the introductory section, the contributions of the paper are highlighted as follows.

- By making progress along the direction of the CZN approach, the paper proposes an effective SEZN approach and its associated controller to promote the convergence properties, and accelerate the synchronization process of chaotic sensor systems.
- The controller designed by the proposed SEZN approach distinctively and inherently possesses the advantage of super-exponential convergence, which makes the synchronization process faster and more accurate. It is a breakthrough in the convergence research of the neurodynamic approach and real-time chaotic synchronization of sensor systems.
- Theoretical analyses on the stability and convergence advantages in terms of both faster convergence speed and lower error bound within the synchronization duration are shown in detail to guarantee the validity and advantage of the SEZN approach and its associated controller.
- Simulation studies including three synchronization examples, comparisons with other methods as well as extensive tests all verify the effectiveness as well as superiority of the SEZN approach and the related controller in practice.

## 2. Preliminaries and Neurodynamic Approaches

The formulation of real-time synchronization between two chaotic sensor systems is presented as preliminaries in this section. Then, the SEZN approach is proposed for the design of the controller. For comparative purposes, the CZN approach is also presented accordingly.

### 2.1. Synchronization of Chaotic Systems

Let us consider a master chaotic system with a general form as follows:

(1) $${\dot{x}}_{m}\left(t\right)=f_{m}\left(x_{m}\left(t\right)\right),$$

where $x_{m}\left(t\right)={\left[x_{m1}\left(t\right),x_{m2}\left(t\right),\cdots ,x_{mn}\left(t\right)\right]}^{T}\in R^{n}$ is a state vector of master chaotic system, and $f_{m}\left(\cdot \right):R^{n}\to R^{n}$ is a nonlinear mapping vector of a specific master chaotic sensor system. Correspondingly, a slave chaotic system with a general form is presented:

(2) $${\dot{x}}_{s}\left(t\right)=f_{s}\left(x_{s}\left(t\right)\right)+u\left(t\right),$$

where vector $x_{s}\left(t\right)={\left[x_{s1}\left(t\right),x_{s2}\left(t\right),\cdots ,x_{sn}\left(t\right)\right]}^{T}\in R^{n}$ is a state vector of slave chaotic system, and mapping $f_{s}\left(\cdot \right):R^{n}\to R^{n}$ is a nonlinear mapping vector of a specific slave chaotic system. In addition, vector $u\left(t\right)={\left[u_{1}\left(t\right),u_{2}\left(t\right),\cdots ,u_{n}\left(t\right)\right]}^{T}\in R^{n}$ is a control-input vector transmitted to a slave chaotic system for synchronization.

If the initial state vectors $x_{m}\left(0\right)$ and $x_{s}\left(0\right)$ of both master as well as slave chaotic systems differ from each other, the state trajectories of such systems differ quite a lot. The objective for synchronization between master chaotic system (1) and slave chaotic system (2) is to develop a control-input vector u(t) so that the slave system is forced to track the master system with the synthesized state error $e\left(t\right)=x_{m}\left(t\right)-x_{s}\left(t\right)$ converging to zero. Extensive research for real-time chaotic synchronization of sensor systems has been proposed by leveraging the CZN approach for fully exploiting the time-derivative information. The associated models and controllers designed by the CZN approach have been proven to possess the global as well as exponential convergence property. However, such a convergence property may not be sufficient for a strict real-time requirement in a complex environment, such as real-time secure communication systems and radar communication systems [57,58]. In addition, as the complexity of systems increases, the computational scale would become intensively large, and the synchronization accuracy would become particularly low when the real-time capability of chaotic systems needs to be considered in practical applications. To tackle the tough issue in the above discussion, we make progress along the direction of CZN research, and further propose a new SEZN approach with super-exponential convergence property.

**Remark** **1.** *Note that most of the specific chaotic systems could be formulated as the master chaotic system (1) with a general form of nonlinear differential equations, such as Lu chaotic systems [53], Chen chaotic systems [55], and Lorenz chaotic systems [56], which covers most common chaotic systems [31].*

### 2.2. Neurodynamic Approaches

The formulation for chaotic synchronization of sensor systems is presented. In this section, we propose the SEZN approach for designing the controller of chaotic sensor systems. For further investigation and better comparison, the controller designed by the CZN approach is also presented. To achieve the real-time synchronization of slave chaotic system (2) and master chaotic system (1) under the influence of external disturbances, a controller is designed by the following SEZN approach.

First, to monitor as well as to control the synchronization procedure of chaotic systems (2) and (1), a vector valued error-function (for the real-time measurement of difference between states of master and slave chaotic sensor systems) is defined as follows:

(3) $$e\left(t\right)=x_{m}\left(t\right)-x_{s}\left(t\right),$$

To make each element $e_{i}\left(t\right)$, with i=1,2,⋯,n, of the synthesized error (3) converge towards zero with respect to time *t*, the SEZN approach is utilized with the following dynamic equation:

(4) $$\dot{e}\left(t\right)=-\lambda \exp\left(t\right)Y\left(e\left(t\right)\right),$$

where design parameter $\lambda \in R^{+}$ is set for the stability and convergence of neurodynamic model. Besides, $Y\left(\cdot \right):R^{n}\to R^{n}$ is an alternative activation function vector mapping with each element being a monotonically-increasing-odd-function, which could be used to accelerate convergence speed of the neurodynamic model. Without loss of generality and for simplicity, a linear activation function Y(e(t))=e(t) is used and investigated in the paper.

By substituting chaotic systems (2) and (1) into dynamic Equation (4), the corresponding neurodynamic model for the chaotic synchronization of sensor systems is depicted as follows:

(5) $$\begin{array}{c} f_{m}\left(x_{m}\left(t\right)\right)-f_{s}\left(x_{s}\left(t\right)\right)-u\left(t\right)=-\lambda \exp\left(t\right)Y\left(x_{m}\left(t\right)-x_{s}\left(t\right)\right) \end{array}$$

with $\dot{e}\left(t\right)={\dot{x}}_{m}\left(t\right)-{\dot{x}}_{s}\left(t\right)$. Due to the unavoidable existence of unexpected external time-varying disturbances in the complex environment, the synchronization of chaotic systems would be a knotty time-varying problem. As readily founded in the neurodynamic model (5), the proposed SEZN approach can effectively handle the disturbance rejection issue in a relatively simple manner via exploiting the time-derivative information as well as the super-exponential properties of the involved chaotic systems.

According to neurodynamic model (5), the associated controller with explicit control-input vector u(t) is thus designed:

(6) $$\begin{array}{c} u\left(t\right)=f_{m}\left(x_{m}\left(t\right)\right)-f_{s}\left(x_{s}\left(t\right)\right)+\lambda \exp\left(t\right)Y\left(x_{m}\left(t\right)-x_{s}\left(t\right)\right). \end{array}$$

**Figure 2:**
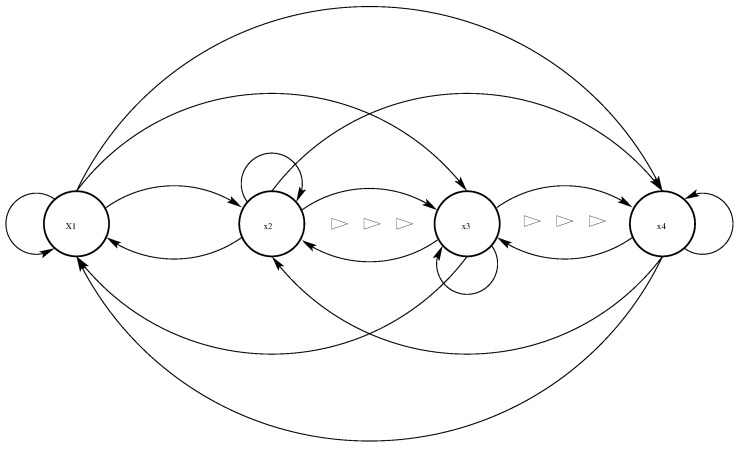
Neuron-connection architecture of the associated RNN model for designing the controller of chaotic sensor systems with $x_{si}$ denoting the *i*th neuron.

Note that the associated controller (6) designed by the proposed SEZN approach does not require any information of external disturbances which is thus applicable in practical applications. The neuron-connection architecture of the RNN model for designing the controller of chaotic systems via the proposed SEZN approach is presented in Figure 2. As one can readily find from the figure, the RNN model in the paper is a typical kind of Hopfield-type RNN [51] with each layer having *n* neurons. Unlike the traditional feed-forward neural networks (FNNs), the RNNs are the neural networks that possess the feedback connections of each network layers [59]. The related structure of RNNs is more complicated than the one of FNNs. More specifically, each neuron in the RNNs exports outputs to other neurons via the connected synapses. At the same time, each neuron in the RNNs receives inputs from other neurons via the connected synapses. The input information depends on the initial states of the RNNs. Then, the real-time states vary adaptively. Finally, the RNNs converge to the equilibrium states (or termed steady states), and the steady states are the outputs of the RNNs. The output signals of the RNN model are transmitted to the effectors, e.g., the slave chaotic sensor systems. For superiorities of parallelism and easy implementation by hardware, the RNN model can be implemented by utilizing embedded systems such as field-programmable gate arrays (FPGAs) [26,27,60]. Many state-of-the-art studies [27,61,62] have been reported for the effective implementation of the neural network model such as the MLP. In [27], the MLP was effectively implemented by utilizing the FPGA Ciclone IV GX FPGA DE2i-150 from Altera. Such an MLP is interfaced with a computer by utilizing the serial communication protocol to feed the input data, which was introduced in detail in [60]. The extensions of the above RNN model to the real implementations on FPGAs could be interesting and open future research directions.

To lay a basis for further investigation and comparison, the controller designed by the CZN approach for the chaotic synchronization of sensor systems is also presented as followed [31]:

(7) $$u\left(t\right)=f_{m}\left(x_{m}\left(t\right)\right)-f_{s}\left(x_{s}\left(t\right)\right)+\gamma \Psi \left(x_{m}\left(t\right)-x_{s}\left(t\right)\right),$$

where design parameter $\gamma \in R^{+}$ is set for the stability and convergence of the above controller, and $\Psi \left(\cdot \right):R^{n}\to R^{n}$ is an alternative activation-function vector mapping for the CZN approach. For fair comparisons, design parameters are selected to be γ=λ, and activation-function vector mappings Y(·) and Ψ(·) are both set to be linear activation function.

## 3. Theoretical Analyses

To confirm the validity and superiority of the proposed SEZN approach and the related controller (6) for the synchronization of chaotic systems, the theoretical analyses are presented in detail. In addition, the corresponding theoretical results of the CZN approach are also presented for better comparison.

**Definition** **1.** *[63] For chaotic synchronization of sensor systems (1) and (2), starting with a random initial state $x_{s}\left(0\right)$, a vector valued error function e(t) at time t≥0 synthesized by a control system is said to be globally and exponentially convergent to zero if it satisfies*

$${∥e\left(t\right)∥}_{E}\leq \alpha {∥e\left(0\right)∥}_{E}\exp\left(-\beta t\right),\;\forall t\geq 0,$$

*where symbol ${∥\cdot ∥}_{E}$ denotes the Euclidean norm of a vector, and constants α and β exist with β being the exponential convergence rate of ${∥e\left(t\right)∥}_{E}$.*

**Definition** **2.** *[63] For the chaotic synchronization of sensor systems (1) and (2), starting with a random initial state $x_{s}\left(0\right)$, a state trajectory $x_{s}\left(t\right)$ of slave chaotic system (2) at time t≥0 synthesized by a control system is said to be globally and exponentially convergent to the state $x_{m}\left(t\right)$ of master chaotic system (1) if it satisfies*

$$∥x_{m}\left(t\right)-x_{s}{\left(t\right)∥}_{E}\leq \alpha {∥x_{m}\left(0\right)-x_{s}\left(0\right)∥}_{E}\exp\left(-\beta t\right).\;\forall t\geq 0.$$

**Definition** **3.** *[64] For the chaotic synchronization of sensor systems (1) and (2), starting with a random initial state $x_{s}\left(0\right)$, a vector-valued error function e(t) at time t≥0 synthesized by a control system is said to be globally and super-exponentially convergent to zero if it satisfies*

$${∥e\left(t\right)∥}_{E}\leq \alpha {∥e\left(0\right)∥}_{E}\exp\left(-\beta \exp\left(t\right)\right),\;\forall t\geq 0,$$

*where constants α and β exist with βexp(t)/t being the super-exponential convergence rate of ${∥e\left(t\right)∥}_{E}$.*

**Definition** **4.** *[64] For the chaotic synchronization of sensor systems (1) and (2), starting with a random initial state $x_{s}\left(0\right)$, a state trajectory $x_{s}\left(t\right)$ of slave chaotic system (2) at time t≥0 synthesized by a control system is said to be globally and super-exponentially convergent to the state $x_{m}\left(t\right)$ of master chaotic system (1) if it satisfies*

$$∥x_{m}\left(t\right)-x_{s}{\left(t\right)∥}_{E}\leq \alpha {∥x_{m}\left(0\right)-x_{s}\left(0\right)∥}_{E}\exp\left(-\beta \exp\left(t\right)\right).\;\forall t\geq 0.$$

**Theorem** **1.** *[65,66] For the chaotic synchronization of sensor systems (1) and (2), starting with a random initial state $x_{s}\left(0\right)$, the control system equipped with controller (6) is globally stable in the sense of Lyapunov.*

**Proof** **of** **Theorem** **1.** For handling the synchronization of chaotic sensor systems (1) and (2), the dynamical equation of the closed-loop control system designed by the proposed SEZN approach is depicted as follows:

(8) $$\dot{e}\left(t\right)=-\lambda \exp\left(t\right)Y\left(e\left(t\right)\right),$$

where $e\left(t\right)=x_{m}\left(t\right)-x_{s}\left(t\right)$. If a linear activation-function processing-array Y(·) is utilized, Equation (8) can be rewritten as

$$\dot{e}\left(t\right)=-\gamma \exp\left(t\right)e\left(t\right).$$

Let us define a Lyapunov function candidate:

$$L\left(t\right)=\frac{{∥e\left(t\right)∥}_{E}^{2}}{2}=\frac{e^{T}\left(t\right)e\left(t\right)}{2}.$$

Note that L(t) is positive-definite in view of L(t)>0 for e(t)≠0, and L(t)=0 for e(t)=0 only. Afterwards, one can have the time-derivative of L(t) as

$$\dot{L}\left(t\right)=\frac{dL(t)}{dt}=e^{T}\left(t\right)\frac{de(t)}{dt}=-\lambda \exp\left(t\right)e^{T}\left(t\right)e\left(t\right).$$

Therefore, one can obtain the result that $\dot{L}\left(t\right)$ is negative-definite for time t∈[0,+∞) with design parameter λ>0. Based on the Lyapunov stability theory [66], the control system equipped with controller (6) is globally stable. The proof is thus completed. □

**Theorem** **2.** *[64,65] For the synchronization of chaotic sensor systems (1) and (2), starting with a random initial state $x_{s}\left(0\right)$, the vector-valued error function e(t) at time t≥0 synthesized by control system equipped with controller (6) globally and super-exponentially converges to zero with the super-exponential convergence rate being λexp(t)/t.*

**Proof** **of** **Theorem** **2.** Note that the *i*th dynamical subsystem corresponding to error function e(t) in neurodynamic model (5) is depicted as

(9) $${\dot{e}}_{i}\left(t\right)=-\lambda \exp\left(t\right)e_{i}\left(t\right)$$

with i=1,2,⋯m. Based on the differential equation theory [67], the solution to (9) is

(10) $$e_{i}\left(t\right)=\frac{e_{i}\left(0\right)}{\exp(-\lambda )}\exp\left(-\lambda \exp\left(t\right)\right).$$

The vector-formed of (10) is thus as

(11) $$e\left(t\right)=\frac{e(0)}{\exp(-\lambda )}\exp\left(-\lambda \exp\left(t\right)\right).$$

Therefore, the residue error is obtained as

(12) $${∥e\left(t\right)∥}_{E}=\sqrt{\sum_{i=1}^{m}\frac{e_{i}^{2}\left(0\right)}{\exp(-2\lambda )}}\exp\left(-\lambda \exp\left(t\right)\right).$$

Equation (12) means that the residue error of the neurodynamic model (5) globally and converges to zero with super-exponential convergence rate as λexp(t)/t.

According to Definition 3, we have the result that the vector-valued error function e(t) synthesized by the control system equipped with controller (6) globally and super-exponentially converges to zero with the super-exponential convergence rate being λexp(t)/t. The proof is thus completed. □

**Corollary** **1.** *[64,65] For the synchronization of chaotic senor systems (1) and (2), starting with a random initial state $x_{s}\left(0\right)$, the state trajectory $x_{s}\left(t\right)$ of slave chaotic system (2) at time t≥0 synthesized by a control system equipped with controller (6) globally and super-exponentially converges to the state $x_{m}\left(t\right)$ of master chaotic system (1).*

**Proof** **of** **Corollary** **1.** It can be generalized from Definition 4 and the proof of Theorem 2. □

For better comparison, corresponding theoretical results of the controller (7) designed by the CZN approach for chaotic synchronization of sensor systems (1) and (2) are also provided as the following lemma [31].

**Lemma** **1.** *[63] For the synchronization of chaotic sensor systems (1) and (2), starting with a random initial state $x_{s}\left(0\right)$, the vector-valued error function e(t) at time t≥0 synthesized by control system equipped with controller (7) exponentially converges to zero with the exponential convergence rate being γ.*

**Proof** **of** **Lemma** **1.** The *i*th dynamical subsystem corresponding to error function e(t) of control system equipped with controller (7) is depicted as

(13) $${\dot{e}}_{i}\left(t\right)=-\gamma e_{i}\left(t\right)$$

with i=1,2,⋯m. On the basis of the differential equation theory [67], the solution to (13) is

(14) $$e_{i}\left(t\right)=e_{i}\left(0\right)\exp\left(-\gamma t\right).$$

The vector-formed of (14) can be obtained:

(15) e(t)=e(0)exp(−γt).

The residue error is obtained as follows:

(16) $${∥e\left(t\right)∥}_{E}={∥e_{i}\left(0\right)∥}_{E}\exp\left(-\gamma t\right).$$

Equation (16) means that the residue error of the control system globally and exponentially converges to zero with exponential convergence rate being λ.

According to Definition 1, we have the result that the vector-valued error function e(t) synthesized by the control system equipped with controller (7) globally and exponentially converges to zero with the exponential convergence rate being λ. The proof is thus completed. □

**Corollary** **2.** *[63] For the synchronization of chaotic sensor systems (1) and (2), starting with a random initial state $x_{s}\left(0\right)$, the state trajectory $x_{s}\left(t\right)$ of slave chaotic system (2) at time t≥0 synthesized by a control system equipped with controller (7) globally and exponentially converges to the state $x_{m}\left(t\right)$ of master chaotic system (1).*

**Proof** **of** **Corollary** **2.** It can be generalized from Definition 2 and the proof of Lemma 1. □

To confirm the fast convergence property of the control system equipped with controller (6), the following theorem is presented in terms of convergence performance of the bound of residual error.

**Theorem** **3.** [64,65] For the synchronization of chaotic sensor systems (1) and (2), starting with the same random initial state $x_{s}\left(0\right)$, the upper bound of residual error $∥e_{\mathrm{SEZN}}{\left(t\right)∥}_{E}$ synthesized by a control system equipped with controller (6) is lower than residual error $∥e_{\mathrm{CZN}}{\left(t\right)∥}_{E}$ synthesized by a control system equipped with controller (7) at the same instance $t^{*}\in \left(0,+\infty \right)$, i.e., $∥e_{\mathrm{SEZN}}\left(t^{*}\right){∥}_{E}<{∥e_{\mathrm{CZN}}\left(t^{*}\right)∥}_{E}$, with the same design parameters λ=γ.

**Proof** **of** **Theorem** **3.** According to residual errors of control systems equipped with controllers (6) and (7), define two function candidates to measure the real-time residual errors via the SEZN approach and the CZN approach respectively as follows:

$$\begin{array}{c} L_{\mathrm{SEZN}}\left(t\right)=\frac{∥e_{\mathrm{SEZN}}{\left(t\right)∥}_{E}^{2}}{2},\\ L_{\mathrm{CZN}}\left(t\right)=\frac{∥e_{\mathrm{CZN}}{\left(t\right)∥}_{E}^{2}}{2}. \end{array}$$

Both $L_{\mathrm{SEZN}}\left(t\right)$ and $L_{\mathrm{CZN}}\left(t\right)$ are positive-definite in view of $L_{\mathrm{SEZN}}\left(t\right)>0$ and $L_{\mathrm{CZN}}\left(t\right)>0$ for $e_{\mathrm{SEZN}}\left(t\right)\neq 0$ and $e_{\mathrm{CZN}}\left(t\right)\neq 0$, and $L_{\mathrm{SEZN}}\left(t\right)=0$ and $L_{\mathrm{CZN}}\left(t\right)=0$ for both $e_{\mathrm{SEZN}}\left(t\right)=0$ and $e_{\mathrm{CZN}}\left(t\right)=0$ only. Then, we can obtain the time-derivatives of $L_{\mathrm{SEZN}}\left(t\right)$ and $L_{\mathrm{CZN}}\left(t\right)$ respectively as follows:

(17) $$\begin{array}{c} {\dot{L}}_{\mathrm{SEZN}}\left(t\right)=-\lambda \exp\left(t\right)e_{\mathrm{SEZN}}^{T}\left(t\right)e_{\mathrm{SEZN}}\left(t\right), \end{array}$$

(18) $$\begin{array}{c} {\dot{L}}_{\mathrm{CZN}}\left(t\right)=-\gamma e_{\mathrm{CZN}}^{T}\left(t\right)e_{\mathrm{CZN}}\left(t\right). \end{array}$$

Compared (17) with (18), for the same parameters λ=γ and the same initial states $e_{\mathrm{SEZN}}\left(t_{\mathrm{ini}}\right)=e_{\mathrm{CZN}}\left(t_{\mathrm{ini}}\right)$ at time $t_{\mathrm{ini}}\in \left[0,+\infty \right)$, we have

(19) $${\dot{L}}_{\mathrm{SEZN}}\left(t_{\mathrm{ini}}\right)<{\dot{L}}_{\mathrm{CZN}}\left(t_{\mathrm{ini}}\right).$$

In the next moment, i.e., $t_{i}ni=t_{i}ni+∆t$ with ∆t→0, for t∈(0,+∞), we can obtain

(20) $$L_{\mathrm{SEZN}}\left(t_{\mathrm{ini}}+∆t\right)=L_{\mathrm{SEZN}}\left(t_{\mathrm{ini}}\right)+∆t{\dot{L}}_{\mathrm{SEZN}}\left(t_{\mathrm{ini}}\right),$$

and

(21) $$L_{\mathrm{CZN}}\left(t_{\mathrm{ini}}+∆t\right)=L_{\mathrm{CZN}}\left(t_{\mathrm{ini}}\right)+∆t{\dot{L}}_{\mathrm{CZN}}\left(t_{\mathrm{ini}}\right).$$

According to (19), with the same initial states, i.e., $e_{\mathrm{SEZN}}\left(t_{\mathrm{ini}}\right)=e_{\mathrm{CZN}}\left(t_{\mathrm{ini}}\right)\neq 0$, we have

$$L_{\mathrm{SEZN}}\left(t_{\mathrm{ini}}+∆t\right)<L_{\mathrm{CZN}}\left(t_{\mathrm{ini}}+∆t\right).$$

Therefore, we have the conclusion that

(22) $$∥e_{\mathrm{SEZN}}\left(t^{*}\right){∥}_{E}<{∥e_{\mathrm{CZN}}\left(t^{*}\right)∥}_{E},$$

where $t^{*}=t_{i}ni+∆t\in \left(0,+\infty \right)$. Equation (22) means that the upper bound of residual error synthesized by a control system equipped with controller (6) is lower than residual error $∥e_{\mathrm{CZN}}{\left(t\right)∥}_{E}$ synthesized by a control system equipped with controller (7) at the same instance $t^{*}\in \left(0,+\infty \right)$ with the same parameters λ=γ. The proof is thus completed. □

## 4. Verifications, Comparisons and Tests

Simulation verifications including three synchronization examples, comparisons with other approaches, and extensive tests are provided to substantiate the validity, fast convergence performance as well as superiority of the proposed SEZN approach and the associated controller (6) for the chaotic synchronization of sensor systems.

### 4.1. Synchronization Examples

In the examples, the synchronization of two identical Lu chaotic systems, two identical autonomous chaotic systems, and two nonidentical chaotic systems are successively considered and presented. Without losing generality, the initial value of each state of chaotic sensor systems is set to be $x_{m}\left(0\right)={\left[1,1,1\right]}^{T}$ and $x_{s}\left(0\right)={\left[3,3,3\right]}^{T}$. The numerical studies are conducted on MATLAB R2014a environment implemented by a personal digital computer with a CPU of Inter(R) Core(TM) i5-7400U @ 3.00 GHz, 4.00 GB memory as well as a Windows 10 Ultimate operating system.

#### 4.1.1. Synchronization of Two Identical Lu Chaotic Systems

Let us investigate the following Lu chaotic system [31]:

(23) $$\left\{\begin{array}{c} {\dot{x}}_{1}\left(t\right)=a\left(x_{2}\left(t\right)-x_{1}\left(t\right)\right),\\ {\dot{x}}_{2}\left(t\right)=-x_{1}x_{3}\left(t\right)+cx_{2}\left(t\right),\\ {\dot{x}}_{3}\left(t\right)=x_{1}\left(t\right)x_{2}\left(t\right)-bx_{3}\left(t\right), \end{array}\right.$$

where a=36, b=3 and c=20. For the synchronization of identical Lu chaotic systems, the real-time master chaotic system can be depicted in

(24) $${\dot{x}}_{m}\left(t\right)=\left[\begin{array}{c} a(x_{m2}\left(t\right)-x_{m1}\left(t\right))\\ -x_{m1}x_{m3}\left(t\right)+cx_{m2}\left(t\right)\\ x_{m1}\left(t\right)x_{m2}\left(t\right)-bx_{m3}\left(t\right) \end{array}\right],$$

and the real-time slave chaotic system with control input vector can be depicted in

(25) $${\dot{x}}_{s}\left(t\right)=\left[\begin{array}{c} a(x_{s2}\left(t\right)-x_{s1}\left(t\right))\\ -x_{s1}x_{s3}\left(t\right)+cx_{s2}\left(t\right)\\ x_{s1}\left(t\right)x_{s2}\left(t\right)-bx_{s3}\left(t\right) \end{array}\right]+u\left(t\right).$$

**Figure 3:**
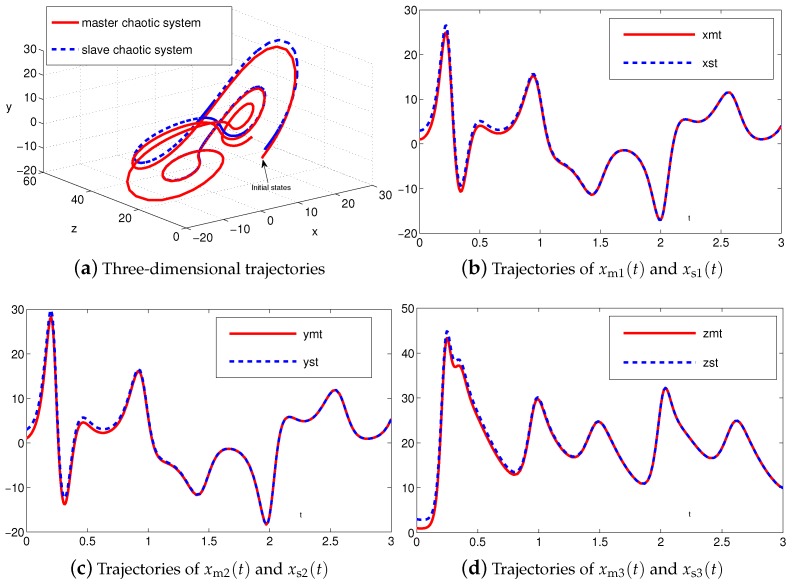
Synchronization performance between two identical Lu chaotic systems (24) and (25) equipped with controller (6) via the proposed super-exponential-zeroing neurodynamic (SEZN) approach.

**Figure 4:**
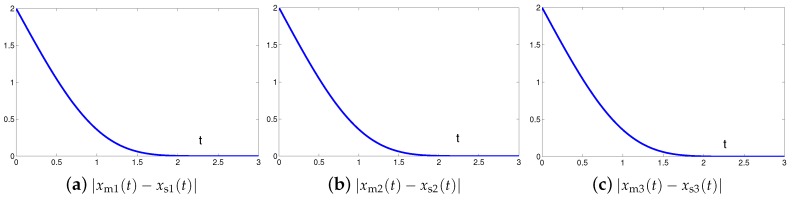
Absolute errors between two identical Lu chaotic systems (24) and (25) equipped with controller (6) via the proposed SEZN approach.

The synchronization duration of this example is set to be $T_{d}=3$ s. In addition, design parameter is λ=1. The corresponding simulation results of synchronization of two identical Lu chaotic systems (24) and (25) equipped with controller (6) using the proposed SEZN approach are illustrated in Figure 3 and Figure 4. Specifically, Figure 3a shows real-time synchronization of two such identical Lu chaotic systems (24) and (25) in three-dimensional space. With different values of initial states, the slave Lu chaotic systems (25) quickly synchronize toward the master Lu chaotic systems (24). In addition, Figure 3b–d respectively illustrate each state, i.e., $x_{s1}$, $x_{s2}$ and $x_{s3}$ of the slave system, which coincides well with each state, i.e., $x_{m1}$, $x_{m2}$ and $x_{m3}$, of the master system. As shown in Figure 4, the absolute values of synchronization errors of all states are relatively small (or say, ignorable), and quickly converge to zero. Moreover, the supremum of each error keeps showing the convergence tendency during the synchronization process, which is consistent with the theoretical result presented in Theorem 2, i.e., with the error function being super-exponentially convergent to zero. The above results illustrate the fast convergence performance of the proposed SEZN approach as well as the associated controller (6) for the synchronization of two identical Lu chaotic systems.

**Figure 5:**
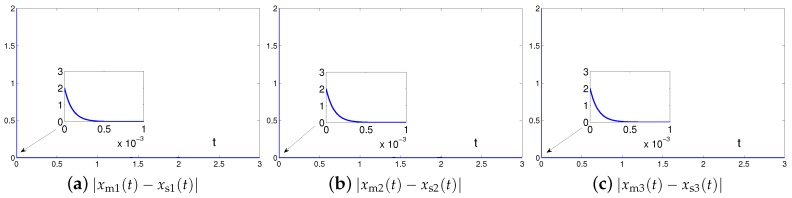
Absolute errors between two identical Lu chaotic systems (24) and (25) equipped with controller (6) via the proposed SEZN approach by choosing design parameter $\lambda =10^{4}$.

It is worth pointing out here that the works in [68,69] implemented on CMOS integrated circuits and FPGA embedded systems possess outstanding synchronization performance between two chaotic systems, and the associated errors are accomplished very fast, i.e., in the very minimum number of iterations. In the illustrative examples of the numerical simulations, the chaotic systems are synchronized within 2.4 s (the synchronization task duration $T_{d}=3$ s) with the design parameter being λ=1. Note that the selection of design parameter λ can be predefined by practitioners. Theoretically, arbitrary value satisfying λ>0 can be set. In practical applications, for the purpose of acceleration of the convergence rate, the value of design parameter λ can be set as appropriately large as the hardware would permit [70]. To further show the fast synchronization of chaotic systems, the tests have been conducted by using a larger value of design parameter λ. The graphical results are shown in Figure 5. As one can readily see in this figure, two identical Lu chaotic systems (24) and (25) can be synchronized within 0.5 ms (the same synchronization task duration being $T_{d}=3$ s) when the design parameter is set to be $\lambda =10^{4}$. As proved in Theorem 2, the control system presents the super-exponential convergence and synchronization property with convergence rate being λexp(t)/t. Therefore, by choosing appropriately large values of design parameter λ, controller (6) designed via the proposed SEZN approach possesses desirable convergence speed, i.e., within milliseconds, for the fast chaotic synchronization of sensor systems in practice.

#### 4.1.2. Synchronization of Two Identical Autonomous Chaotic Systems

Let us investigate the following new autonomous chaotic system proposed in [14]:

(26) $$\left\{\begin{array}{c} {\dot{x}}_{1}\left(t\right)=p\left(x_{2}\left(t\right)-x_{1}\left(t\right)\right)+x_{2}\left(t\right)x_{3}\left(t\right),\\ {\dot{x}}_{2}\left(t\right)=\left(r-p\right)x_{1}-x_{1}x_{3}\left(t\right)+rx_{2}\left(t\right),\\ {\dot{x}}_{3}\left(t\right)=-qx_{3}\left(t\right)-sx_{2}\left(t\right)x_{2}\left(t\right), \end{array}\right.$$

where p=40, q=5, r=30 and s∈[0,10]. For synchronization of identical autonomous chaotic systems with the above form, the real-time master chaotic system is described as

(27) $${\dot{x}}_{m}\left(t\right)=\left[\begin{array}{c} p\left(x_{m2}\left(t\right)-x_{m1}\left(t\right)\right)+x_{m2}\left(t\right)x_{m3}\left(t\right)\\ \left(r-p\right)x_{m1}-x_{m1}x_{m3}\left(t\right)+rx_{m2}\left(t\right)\\ -qx_{m3}\left(t\right)-sx_{m2}\left(t\right)x_{m2}\left(t\right) \end{array}\right],$$

and the slave chaotic system with control input vector is described as

(28) $${\dot{x}}_{s}\left(t\right)=\left[\begin{array}{c} p\left(x_{s2}\left(t\right)-x_{s1}\left(t\right)\right)+x_{s2}\left(t\right)x_{s3}\left(t\right)\\ \left(r-p\right)x_{s1}-x_{s1}x_{s3}\left(t\right)+rx_{s2}\left(t\right)\\ -qx_{s3}\left(t\right)-sx_{s2}\left(t\right)x_{s2}\left(t\right) \end{array}\right]+u\left(t\right).$$

**Figure 6:**
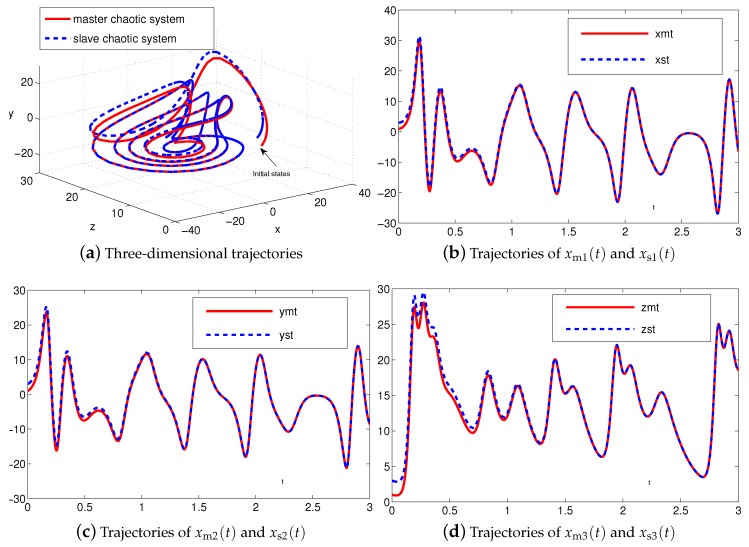
Synchronization performance between two identical autonomous chaotic systems (27) and (28) equipped with controller (6) via the proposed SEZN approach.

**Figure 7:**
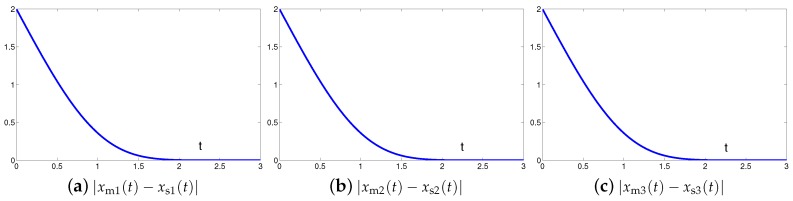
Absolute errors between two identical autonomous chaotic systems (27) and (28) equipped with controller (6) via the proposed SEZN approach.

The synchronization duration of this example is set to be $T_{d}=3$ s. In addition, design parameter is still set to be λ=1. The corresponding simulation results of synchronization of two identical autonomous chaotic systems (27) and (28) equipped with controller (6) using the proposed SEZN approach are illustrated in Figure 6 and Figure 7. Specifically, Figure 6a shows real-time synchronization of two such identical autonomous chaotic systems (27) and (28) in three-dimensional space. With different values of initial states, the slave autonomous chaotic systems (28) also quickly synchronize toward the master autonomous chaotic systems (27). In addition, Figure 6b–d respectively show each state, i.e., $x_{s1}$, $x_{s2}$ and $x_{s3}$ of the slave system, coincides well with each state, i.e., $x_{m1}$, $x_{m2}$ and $x_{m3}$, of the master system within the synchronization duration. As we can see in Figure 7, the absolute values of synchronization errors of all states are also relatively small (or say, ignorable), and quickly converge to zero. Moreover, the supremum of each error shows super-exponential convergence property. The error function is super-exponentially convergent toward zero. The above results also illustrate the fast convergence performance of the proposed SEZN approach as well as the associated controller (6) for the synchronization of two identical autonomous chaotic systems.

To further show the performance details (i.e., quantifying) of the rejection of chaotic disturbances, the absolute values of position errors, i.e., $|e_{i}\left(t\right)|$, synthesized by the proposed SEZN approach and its associated controller at different time instants, i.e., t=0.6 s, 1.2 s, 1.8 s, 2.4 s and 3 s, in three synchronization examples are presented in Table 1, Table 2 and Table 3, respectively. As one can readily find in the graphical results, the absolute values of errors show similar convergence properties for rejection of chaotic disturbances. The errors show fast decreasing tendency, and rapidly converge towards zero with the global and super-exponential properties. Such graphical results are also consistent with theoretical analyses in Section 3.

Moreover, we have summarized number of iterations and estimation time for synchronization of chaotic systems in the above three synchronization examples (i.e., two identical Lu chaotic systems, two identical autonomous chaotic systems, and two nonidentical chaotic systems), and presented the associated graphical results in Table 4. As shown in Table 4, if the step size is selected to be h=0.001 and the design parameter is set to be λ=1, each estimation time for synchronization in three examples is less then 2.4 s with the number of iterations being less than 2400.

#### 4.1.3. Synchronization of Two Nonidentical Chaotic Systems

We further investigate and achieve synchronization of nonidentical chaotic systems, i.e., with the maser chaotic system being the Lu chaotic system (24) and the slave chaotic system being the autonomous chaotic system (28).

**Figure 8:**
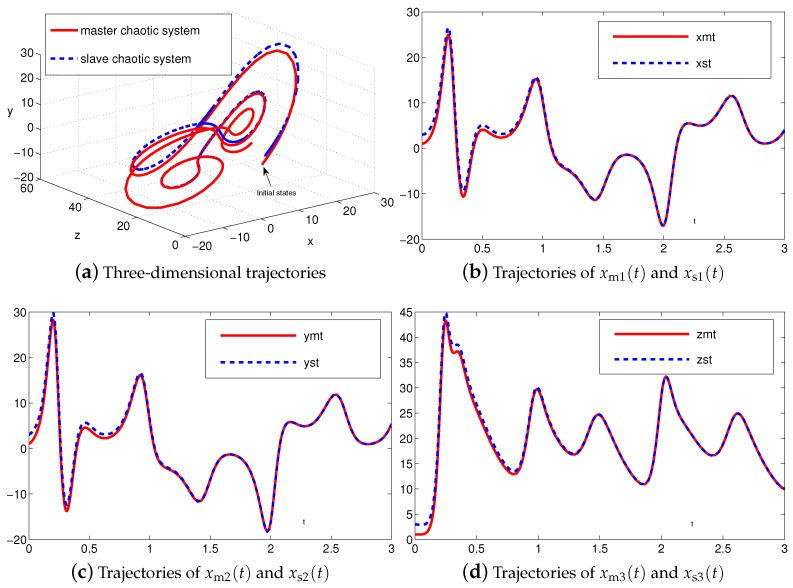
Synchronization performance between two nonidentical chaotic systems (24) and (28) equipped with controller (6) via the proposed SEZN approach.

**Figure 9:**
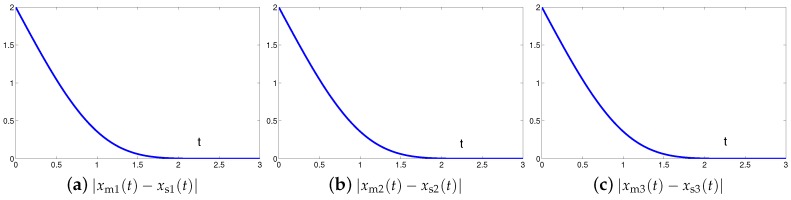
Absolute errors between two nonidentical chaotic systems (24) and (28) equipped with controller (6) via the proposed SEZN approach.

The synchronization duration of this example is set to be $T_{d}=3$ s. In addition, design parameter is set to be λ=1. The corresponding simulation results of synchronization of two nonidentical chaotic systems (24) and (28) equipped with controller (6) using the proposed SEZN approach are illustrated in Figure 8 and Figure 9. Specifically, the real-time synchronization of two such nonidentical chaotic systems (24) and (28) is shown in Figure 8a in three-dimensional space. With different initial states, the slave autonomous chaotic system (28) still quickly synchronizes toward the master Lu chaotic system (24). In addition, Figure 8b–d respectively show each state, i.e., $x_{s1}$, $x_{s2}$ and $x_{s3}$ of the slave autonomous chaotic system (28), almost overlaps each state, i.e., $x_{m1}$, $x_{m2}$ and $x_{m3}$, of the master Lu chaotic system (24) within the synchronization duration. As shown in Figure 9, the absolute values of synchronization errors of all states are also relatively small (or say, ignorable), and quickly converge to zero. Moreover, the supremum of each error shows super-exponential convergence property. The error function is super-exponentially convergent to zero, for the case of synchronization of two nonidentical chaotic systems. The above results also illustrate the fast convergence performance of the proposed SEZN approach as well as the associated controller (6) for the synchronization of two nonidentical chaotic systems.

### 4.2. Comparisons with Other Approaches

In this subsection, to substantiate the fast convergence property and superiority of the proposed SEZN approach, we conduct and show the detailed comparisons by using other approaches, e.g., the CZN approach, for the synchronization of chaotic systems under the same conditions.

**Figure 10:**
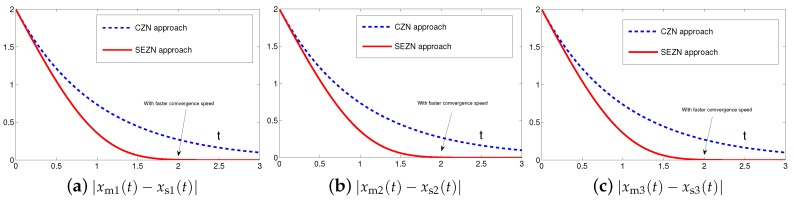
Absolute errors between two identical Lu chaotic systems (24) and (25) equipped with controller (7) via the CZN approach in comparison with controller (6) via the proposed SEZN approach.

**Figure 11:**
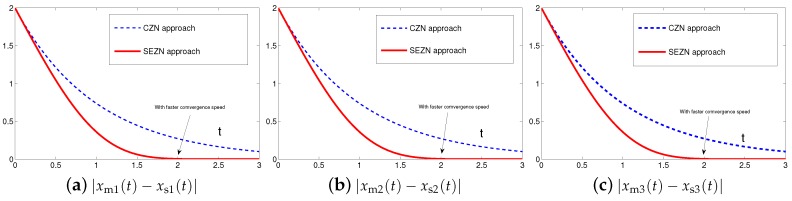
Absolute errors between two identical autonomous chaotic systems (27) and (28) equipped with controller (7) via the CZN approach in comparison with controller (6) via the proposed SEZN approach.

**Figure 12:**
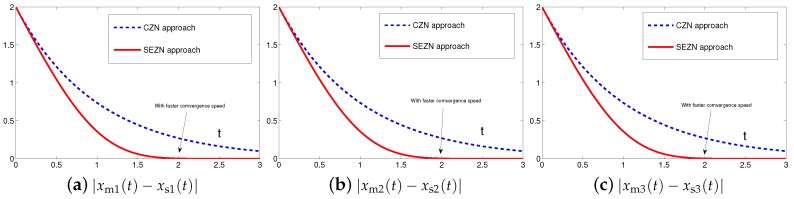
Absolute errors between two nonidentical chaotic systems (24) and (28) equipped with controller (7) via the CZN approach in comparison with controller (6) via the proposed SEZN approach.

Originally derived from Zhang et al. [31] to design the neurodynamic models, the CZN approach is able to handle different time-varying problems including the synchronization of chaotic systems. Specifically, for synchronization of chaotic systems, the associated controller designed by the CZN approach is depicted as (7). For better comparison, the design parameter of the CZN approach is set to be γ=λ=1 in this simulation. In addition, other simulation conditions are set the same as those in Section 4.1. The comparative simulation results of synchronization performance between two identical Lu chaotic systems (24) and (25), two identical autonomous chaotic systems (27) and (28) and two nonidentical chaotic systems (24) and (28) equipped with controllers (6) and (7) via the proposed SEZN and CZN approaches are presented in Figure 10, Figure 11 and Figure 12. Specifically, in three comparative synchronization examples, the residual errors synthesized by two controllers designed by SEZN and CZN approach, possess different convergence properties, i.e., super-exponential convergence and exponential convergence properties. As shown in these figures, the residual error synthesized by controller (6) designed by the proposed SEZN approach present faster convergence speed in transient state and lower error bound in steady state compared with that of synthesized by controller (7) designed by the CZN approach. The above comparative results are consistent with the theoretical results presented in Theorem 3.

Moreover, to further show the advantages of the controller (6) designed by the proposed SEZN approach, comprehensive comparisons among different approaches for the synchronization of chaotic systems are summarized in Table 5. As seen and compared from the table, the SEZN proposed in this paper possesses the fast convergence speed, i.e., the super-exponential convergence, and global convergence property, which is substantiated via three examples in Section 4.1. In addition, there is no parameter selection limitation during the design process of the control system. Compared with other approaches [14,31,53,54,55,56,71], the above-mentioned advantages make the controller (6) designed by the proposed SEZN approach more suitable for practical applications of the real-time synchronization of chaotic systems with the requirement of fast computational speed.

### 4.3. Extensive Tests

**Figure 13:**
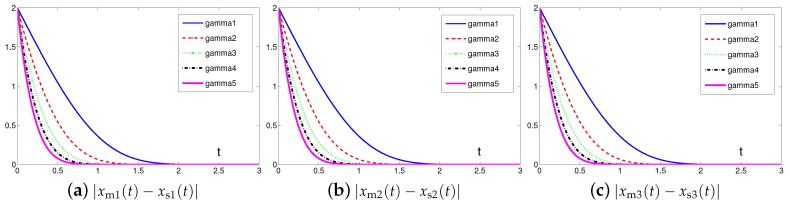
Absolute errors between two identical Lu chaotic systems (24) and (25) equipped with controller (6) via the proposed SEZN approach using different values of design parameter λ.

**Figure 14:**
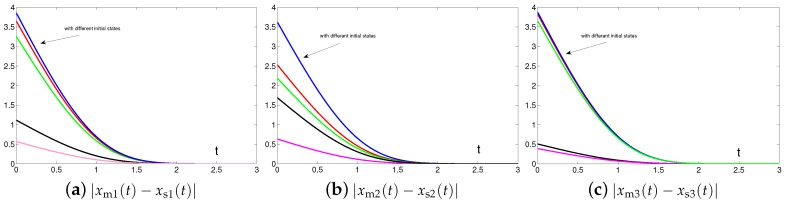
Absolute errors between two identical Lu chaotic systems (27) and (28) equipped with controller (6) via the proposed SEZN approach using randomly generated initial states.

To investigate the synchronization and convergence performance of the controller (6) designed by the proposed SEZN approach, extensive tests in terms of residual error under the conditions of different values of design parameter λ and different values of initial states $x_{m}\left(0\right)$ and $x_{s}\left(0\right)$ of master and slave chaotic systems are conducted respectively. The corresponding test results are shown in Figure 13 and Figure 14. Firstly, we investigate the impact on the convergence performance of the selection of design parameter λ. As shown in Figure 13, starting from the same initial states, the residual errors present faster convergence tendency as the values of design parameter λ gradually increase from 1 to 5. In other words, the convergence speed of residual errors synthesized by controller (6) designed via the proposed SEZN approach can further be improved by increasing the design parameter appropriately. Such graphical results are also consistent with the theoretical results presented in Theorem 2. That is to say, the control system presents the super-exponential convergence and synchronization property with convergence rate being λexp(t)/t. In practical applications, by choosing appropriate values of design parameter λ, controller (6) designed via the proposed SEZN approach possesses desirable convergence speed for the real-time synchronization of chaotic systems.

Then, we investigate the impact of initial states $x_{m}\left(0\right)$ and $x_{s}\left(0\right)$ of master and slave chaotic systems on the convergence performance. As shown in Figure 14, from five different randomly generated initial states $x_{m}\left(0\right)$ and $x_{s}\left(0\right)$, the residual errors synthesized by controller (6) designed via the proposed SEZN approach present similar convergence property, i.e., similar convergence speed in transient state and similar error bound in steady state. Such graphical results are also consistent with the theoretical results presented in Theorem 2. That is to say, the residual errors synthesized by controller (6) designed via the proposed SEZN approach converge to zero globally.

All results verify that the SEZN approach possesses the outstanding and fast convergence properties for the real-time synchronization of chaotic systems, and thus is more suitable for practical applications with the requirement of fast computational speed.

**Remark** **2.** *Note that the extensive tests in the above are conducted in terms of synchronization performance and residual error under the conditions of different values of design parameter λ (related to step sizes) and different values of initial states $x_{m}\left(0\right)$ and $x_{s}\left(0\right)$ of master and slave chaotic systems. The tests have been justified to guarantee chaotic regime, i.e., the synchronization performance and residual error. If bad initial conditions or step sizes are chosen, the chaotic behavior would be suppressed in the very short time when the chaotic oscillators or systems are implemented into an FPGA in engineering applications [68,72,73]. Note that many effective remedial strategies, e.g., the trigonometric polynomial method [73], for handling this issue have been proposed and developed. The investigations on the limitation of numerical simulation and possible extension to the real implementation could be interesting and open future research directions.*

## 5. Conclusions and Future Work

In this paper, a novel SEZN approach together with its associated controller (6) has been proposed to improve the convergence performance and accelerate the synchronization process of chaotic sensor systems by rejecting the chaotic disturbances. Superior to the CZN approach with exponential convergence property, the controller designed by the proposed SEZN approach has inherently shown the advantage of super-exponential convergence, which has made the synchronization process faster and more accurate. Theoretical analyses on the stability and convergence advantages in terms of both the faster convergence speed and lower error bound within the duration have been rigorously presented. Three synchronization examples have verified the effectiveness of the proposed SEZN approach and its associated controller for the synchronization of chaotic systems. Comparisons with other approaches, e.g., the CZN approach, have illustrated the convergence superiorities of the proposed SEZN approach. Extensive tests have shown in detail the impact on convergence performance by choosing different values of design parameter and initial state.

Note that the RNN model equipped with a linear activation function thus constitutes a linear controller. Therefore, the theoretical analyses are presented in the framework of the proposed SEZN approach as well as its linear controller. Actually, a nonlinear activation function, such as bi-exponential activation function [74] and Li activation function [40], can also be applied to the design of the controller. The detailed investigation of the theory of the proposed SEZN approach as well as its associated controller in the nonlinear case is generally considered to be a challenging issue. Moreover, many state-of-the-art applications on security applying chaotic systems were implemented on the analog integrated circuits, e.g., the complementary-metal-oxide-semiconductor (CMOS) integrated circuits [69] and digital integrated circuits, e.g., the FPGA embedded systems [68]. Therefore, future work lies in the following facts: (i) detailed investigation of the theory of the proposed SEZN approach and the corresponding controller in nonlinear case; (ii) extension of the proposed controller to the real circuits and systems such as CMOS and FPGA; As a final remark of this paper, to the best of the authors’ knowledge, this is the first work in the framework of zeroing neurodynamic that is able to elegantly accelerate the synchronization process of chaotic systems with the super-exponential convergence property (a fast convergence speed).

## Figures and Tables

**Table 1:** Absolute values of errors between two identical Lu chaotic systems (24) and (25) equipped with controller (6) via the proposed SEZN approach at different time instants during the rejection of chaotic disturbances.

State of Chaotic Systems	$|e_{i}\left(0.6\right)|$	$|e_{i}\left(1.2\right)|$	$|e_{i}\left(1.8\right)|$	$|e_{i}\left(2.4\right)|$	$|e_{i}\left(3\right)|$
Element 1 of (24) and (25)	1.417 s	0.469 s	0.047 s	$8.830\times 10^{-4}$ s	$1.013\times 10^{-8}$ s
Element 2 of (24) and (25)	1.417 s	0.469 s	0.051 s	$9.791\times 10^{-4}$ s	$1.013\times 10^{-8}$ s
Element 3 of (24) and (25)	1.417 s	0.469 s	0.047 s	$8.830\times 10^{-4}$ s	$1.013\times 10^{-8}$ s

**Table 2:** Absolute values of errors between two identical autonomous chaotic systems (27) and (28) equipped with controller (6) via the proposed SEZN approach at different time instants during the rejection of chaotic disturbances.

State of Chaotic Systems	$|e_{i}\left(0.6\right)|$	$|e_{i}\left(1.2\right)|$	$|e_{i}\left(1.8\right)|$	$|e_{i}\left(2.4\right)|$	$|e_{i}\left(3\right)|$
Element 1 of (27) and (28)	1.242 s	0.269 s	0.016 s	$9.082\times 10^{-5}$ s	$1.027\times 10^{-8}$ s
Element 2 of (27) and (28)	1.242 s	0.269 s	0.016 s	$9.082\times 10^{-5}$ s	$1.033\times 10^{-8}$ s
Element 3 of (27) and (28)	1.227 s	0.258 s	0.014 s	$7.811\times 10^{-5}$ s	$1.028\times 10^{-8}$ s

**Table 3:** Absolute values of errors between two nonidentical chaotic systems (24) and (28) equipped with controller (6) via the proposed SEZN approach at different time instants during the rejection of chaotic disturbances.

State of Chaotic Systems	$|e_{i}\left(0.6\right)|$	$|e_{i}\left(1.2\right)|$	$|e_{i}\left(1.8\right)|$	$|e_{i}\left(2.4\right)|$	$|e_{i}\left(3\right)|$
Element 1 of (24) and (28)	1.417 s	0.469 s	0.051 s	$9.791\times 10^{-4}$ s	$1.014\times 10^{-8}$ s
Element 2 of (24) and (28)	1.417 s	0.469 s	0.051 s	$9.791\times 10^{-4}$ s	$1.013\times 10^{-8}$ s
Element 3 of (24) and (28)	1.417 s	0.469 s	0.051 s	$9.791\times 10^{-4}$ s	$1.013\times 10^{-8}$ s

**Table 4:** Number of iterations and estimation time for synchronization of chaotic systems in three synchronization examples.

State of Chaotic Systems	Number of Iterations	Estimation Time for Synchronization ◊
Element 1 of (24) and (25)	2377	2.377 s
Element 2 of (24) and (25)	2378	2.378 s
Element 3 of (24) and (25)	2377	2.377 s
Element 1 of (27) and (28)	2191	2.191 s
Element 2 of (27) and (28)	2191	2.191 s
Element 3 of (27) and (28)	2190	2.190 s
Element 1 of (24) and (28)	2377	2.377 s
Element 2 of (24) and (28)	2377	2.377 s
Element 3 of (24) and (28)	2377	2.377 s

Note of ◊: Estimation time for synchronization denotes the time required for absolute value of synchronization error of each element $|e_{i}\left(t\right)|$ synthesized by the proposed controller being less than $10^{-3}$.

**Table 5:** Comparisons among different approaches for synchronization of chaotic systems.

Approach	Synchronization Speed	Convergence Property	Parameter Limitation
Proposed SEZN	**Super-exponential**	Global	No
CZN	Exponential	Global	No
[14]	Asymptotic	Global	Yes
[53]	Exponential	Global	No
[55]	Exponential	Global	No
[56]	Exponential	Global	No
[71]	Exponential	Global	No
